# Polarization-selective reconfigurability in hybridized-active-dielectric nanowires

**DOI:** 10.1126/sciadv.abn9459

**Published:** 2022-06-15

**Authors:** June Sang Lee, Nikolaos Farmakidis, C. David Wright, Harish Bhaskaran

**Affiliations:** 1 Department of Materials, University of Oxford, Oxford, UK.; 2 Department of Engineering, University of Exeter, Exeter, UK.

## Abstract

Wavelength and polarization are two fundamental properties of light within which information can be encoded and (de)multiplexed. While wavelength-selective systems have widely proliferated, polarization-addressable active photonics has not seen notable progress, primarily because tunable and polarization-selective nanostructures have been elusive. Here, we introduce hybridized-active-dielectric (HAD) nanowires to achieve polarization-selective tunability. We then demonstrate the ability to use polarization as a parameter to selectively modulate the conductance of individual nanowires within a multi-nanowire system. By using polarization as the tunable vector, we show matrix-vector multiplication in a nanowire device configuration. While our HAD nanowires use phase-change materials as the active material, this concept is readily generalized to other active materials hybridized with dielectrics and thus has the potential in a broad range of applications from photonic memories and routing to polarization-multiplexed computing.

## Teaser

Hybridized-active-dielectric (HAD) nanowires can selectively switch based on polarization of light.

## INTRODUCTION

Polarization, one of the fundamental properties of electromagnetic waves, provides extra physical dimensions for multiplexing optical information, enabling applications such as multidimensional spectroscopy (*1*), high-capacity communication (*2*–*4*), ultrafast detection/modulation (*5*, *6*), polarization-controlled holography (*7*), and others (*8*, *9*). However, such multiplexing properties have been mostly passive or transient thus far and have not been used to deliver active modulators or reconfigurable elements in optical systems. Reconfigurable photonics, on the other hand, has been rapidly growing. The use of phase-change materials is established as a functional block for nonvolatile photonic and mixed-mode (optical-electrical) memories (*10*–*15*) with prospective applications in neuromorphic and in-memory computing paradigms (*16*–*18*). Such applications rely on devices that incorporate phase-change materials as the reconfigurable elements and have been shown to achieve nanosecond switching performance with large optical modulation (*11*, *19*) over a large bandwidth.

Reconfiguration in phase-change photonic devices is commonly achieved by controlling the intensity (*12*, *20*, *21*) or pulse shape (*22*) of an incident light wave to switch the phase state of the phase-change material, while resonant structures (*12*) are broadly used to achieve wavelength-selective operation and enhanced modulation depths. The equivalent functionality in polarization-space is, however, absent. This limits substantially the addressability of distinct elements in cascaded systems.

**Fig. 1.**
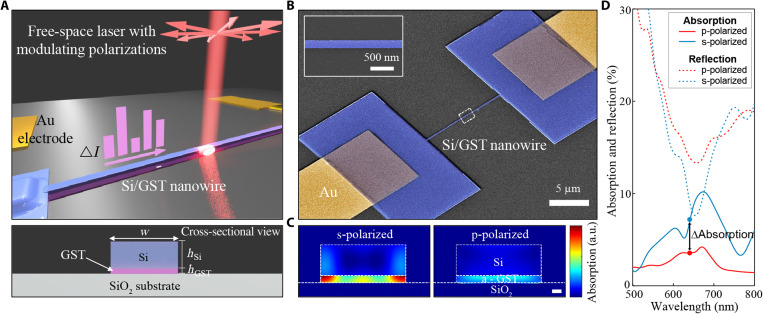
Polarization-selective switching in a HAD nanowire. (**A**) Schematic (top) of the experiment and the cross section (bottom) of our HAD nanowire. Polarized laser illumination (λ = 638 nm) at varying polarizations is shone onto the nanowire, while electrical current (△*I*) is monitored. Nanowire width is ~180 nm, and layer thicknesses are 15 and 65 nm for GST and Si, respectively. (**B**) Colorized SEM images of the device. Inset shows an enlarged view of a nanowire. (**C**) Cross-sectional view of FDTD simulations of absorbed power distribution under s-polarized (left) and p-polarized (right) illumination at 638 nm. Scale bar, 20 nm. a.u., arbitrary units. (**D**) Calculated absorption and reflection spectra of a hybrid nanowire under s-polarized (blue) and p-polarized (red) illumination. Absorption of only the GST layer is considered, and distinct contrast is observed at 638 nm.

Here, we demonstrate polarization-selective switching in hybridized-active-dielectric (HAD) nanowires, wherein the optical absorption in the nanowires is tuned on the basis of the polarization of input optical pulses. The active material that we use is the phase-change material, Ge_2_Sb_2_Te_5_ (GST), and silicon (Si) acts, for the purposes of this work, as our dielectric. Our active material undergoes reversible switching, and by using the hybridized resonant structure, we can tune the absorption of this active layer based on the polarization. Our prototype devices are composed of thin films of Si/GST as shown in Fig. 1 and demonstrate selective excitation and resonant absorption that depends on the direction of incident polarization. Optical isolation between nanowires is achieved by changing their relative orientation to the input polarization. By electrically interfacing the individual nanowires, we further demonstrate electrically coupled yet polarization-decoupled performance, enabling, in this way, matrix-vector multiplications with polarization.

## RESULTS

The schematics and operational principles are illustrated in Fig. 1A, where a free-space optical pump is used to control the crystalline state of the HAD nanowire. A nanowire of 15 μm length and 180 nm width with a combined thickness of 80 nm (15 nm of GST at the bottom with 65 nm of Si on top) is connected between two metal electrodes (detailed fabrication parameters are described in Methods). This allows us to measure the electric current through the GST while being illuminated by the laser. The power and polarization of the laser are modulated during the measurement at a fixed wavelength of 638 nm, for which the nanowires are optimized (figs. S1 and S2). By sending power- and polarization-modulated laser pulses onto a nanowire, the phase of the GST is switched with corresponding changes in electrical resistance from the highly resistive (amorphous) state to the conductive (crystalline) state. Scanning electron microscope (SEM) scans of the device are shown in Fig. 1B.

The choice of Si and GST arises from the requirement of implementing high-index, low-loss resonators (Si) in close contact with a thin switchable absorber (GST). Incoming free-space electromagnetic waves are coupled to high-index dielectric nanowires to induce polarization-dependent photonic resonances (*23*). The position and intensity of the absorption peaks are dictated by nanowire geometry and polarization (*24*, *25*). When a thin layer of high-loss material is introduced below the dielectric cavity, most of the coupled multipole field is asymmetrically absorbed by the lossy layer (figs. S1 and S2) (*26*), where the absorbing layer must remain thin to limit the degradation of the dielectric resonance. This is shown in Fig. 1 (C and D), where the overall absorption within the GST layer is modulated by polarization-selective dielectric resonances of the Si cavity, by which a GST phase transition can be triggered. Figure 1D shows the reflection/absorption spectra of a HAD nanowire at two orthogonal polarizations, calculated by finite-difference time-domain (FDTD). This indicates that, at ~638 nm, the absorption peaks and corresponding reflection dips are sharper when the electric field of the incident light is along the long axis of a nanowire (s-polarized) than when it is oriented perpendicularly to the long axis of the nanowire (p-polarized). At the near-resonant wavelength (638 nm), the Si layer acts as a dielectric resonator, allowing strong modes to be locally confined (fig. S3). These higher-order mode excitations are only observed under s-polarized illumination. However, the response is suppressed under p-polarized illumination, because the perpendicular response of electric/magnetic multipoles vanishes when the nanowire width is much smaller than the wavelengths (*23*, *27*). Such polarization-selective coupling leads to the polarization-dependent absorption within the bottom lossy GST (Fig. 1C).

**Fig. 2.**
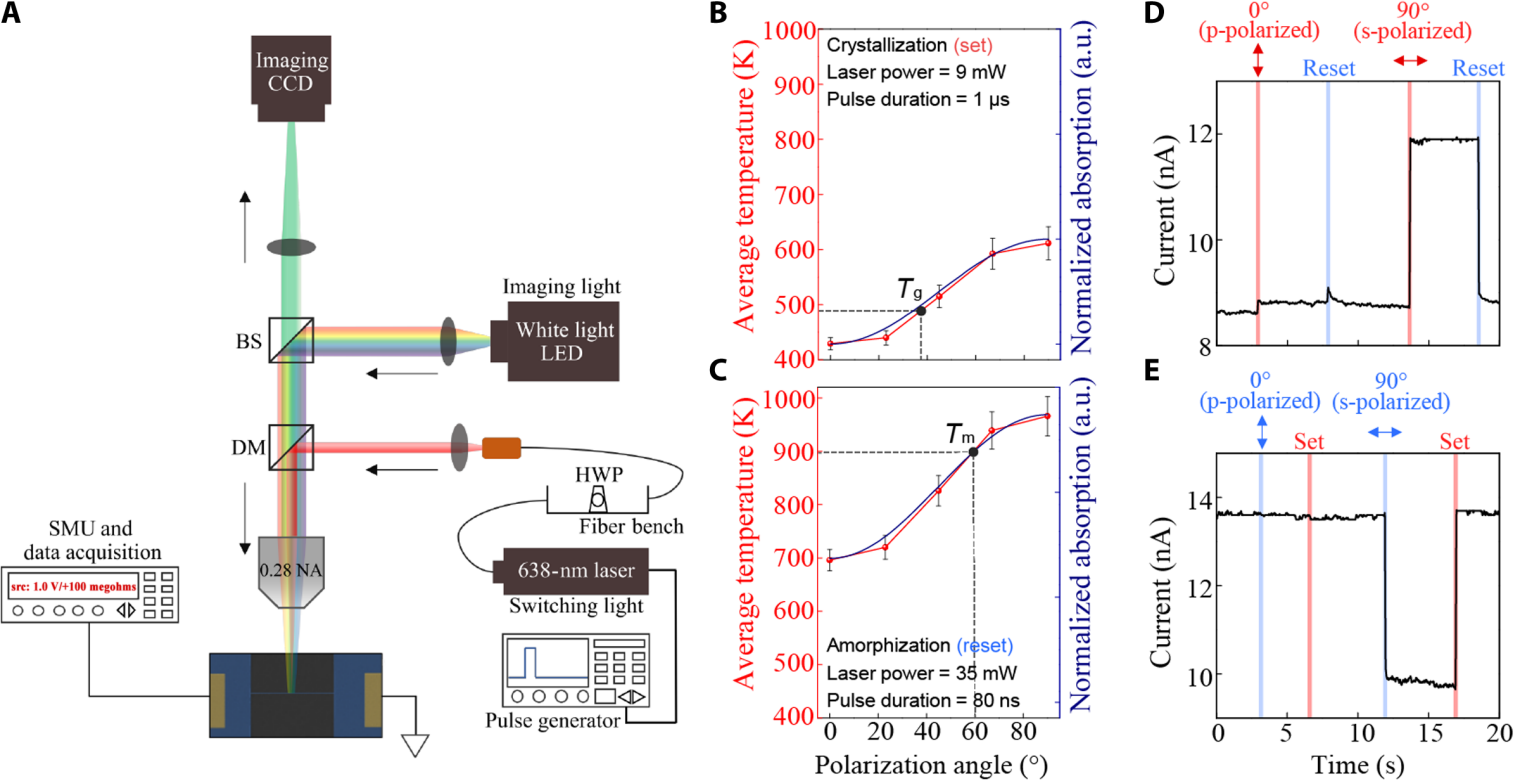
A polarization-selective electro-optic cell. (**A**) Electro-optic measurement schematics. Electrical current across nanowires is monitored, while incident polarization is varied. (**B** and **C**) Calculated average temperature and normalized absorption within the GST of a hybrid nanowire as a function of polarization for (B) crystallization and (C) amorphization. *T*_g_ and *T*_m_ refer to the glass transition temperature and melting point of GST, respectively. The average temperature is calculated from the temperature profile of the nanowire cross section after each pulse. (**D** and **E**) Time-trace measurements of polarization-selective electrical readouts under p-polarized (0°) and s-polarized (90°) pulses for (D) crystallization and (E) amorphization.

We proceed to experimentally evaluate the switching characteristics of our devices by monitoring the conductance state of an initially crystalline nanowire electrically. We apply a 1-V bias between the ends of the nanowire and monitor the resulting current through the nanowire using a source meter unit while simultaneously sending nanosecond-polarized optical pulses at 638 nm onto the device through a 0.28–numerical aperture (NA) objective (Fig. 2A).

Two-dimensional (2D) finite element method (FEM) simulation (COMSOL Multiphysics) is used to determine the optimized conditions for pulse illumination. Details are in the Supplementary Materials (figs. S4 and S5) and Fig. 2 (B and C). From there, we observe that, for both crystallization and amorphization, s-polarized pulses (i.e., 90°) can induce the temperature rise of GST above its glass transition temperature (*T*_g_; ~493 K) and its melting point (*T*_m_; ~900 K), while p-polarized illumination (i.e., 0°) is not able to switch the crystalline states.

Time-trace electrical measurements are shown in Fig. 2 (D and E) for switching pulses at two polarizations (0° and 90°). Because the phase of the GST is completely reversible (fig. S6) (*28*), the state in which we start a set of experiments is at our discretion. For the experiments in Fig. 2, we start in the amorphous phase. Only s-polarized pulses tune the state of nanowires and induce a reversible, nonvolatile change in conductivity. P-polarized illumination induces negligible changes. The change in electrical current (Δ*I*) under s-polarized pulses is observed to be several orders larger than for the case of p-polarized pulses, thereby demonstrating the ability to selectively modulate the electrical characteristics of the nanowire based on the polarization of incident light pulses.

**Fig. 3.**
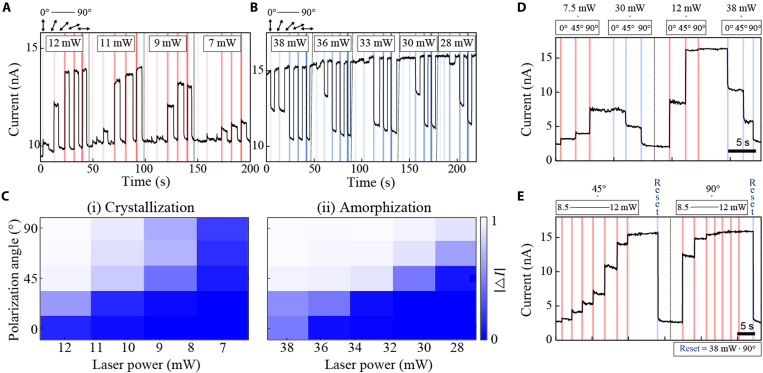
Polarization and power-dependent multilevel modulation. Time-trace electrical readouts for (**A**) crystallization and (**B**) amorphization processes as a function of polarizations from 0° to 90° at each division (colored vertical lines). Laser power varies from 12 to 7 mW (A) (crystallization, red lines) and from 38 to 28 mW (B) (amorphization, blue lines). (**C**) 2D normalized intensity map of measured readout contrasts as a function of polarizations and laser power for (i, left) crystallization and (ii, right) amorphization operations. Freely accessible multilevel operation (**D**) with varying polarizations (0° to 90°) at two different laser powers (7.5 and 12 mW for crystallization, red; and 30 and 38 mW for amorphization, blue) and (**E**) with ramping up the laser power (8.5 to 12 mW) at two different polarizations (45° and 90°). Reset pulse is 38 mW at 80 ns.

We then investigate the ability to tune the conductance of the nanowires between multiple levels by a combination of light intensity modulation (figs. S7 and S8) and polarization selection. A half–wave plate (HWP) is used to control the direction of linearly polarized light, and the power calibration curves used as a function of HWP angles are shown in fig. S9. Five pulses (0° to 90°) are sent for four distinct powers (12 to 7 mW) in Fig. 3A. For 12 mW, we observe that the change in conductance is trivial at 0° polarization and increases rapidly as the polarization approaches to 90°. An equivalent switching threshold behavior is observed for all laser powers tested. In the case of 12 mW, the current begins to increase rapidly at 23° polarization and is saturated at 45° polarization. When the laser power is decreased, the threshold shifts to a higher polarization angle.

An analogous switching behavior is also observed during the amorphization (reset) process. Figure 3B shows the shift of threshold polarization from 0° to 45°, as the reset laser power is reduced from 38 to 28 mW. We also observe that the saturating polarizations increase with decreasing power. This allows us to reach intermediate levels by only controlling polarization, thus providing an additional degree of control in reaching a predetermined conductance state. Figure 3C shows a map the normalized magnitude of readout current changes (∣Δ*I*∣) as a function of incident power and polarizations. The change of readout current becomes saturated at the top left corner when either laser power or polarization is above the threshold. Below the saturation, the conductance state reached varies as a function of both laser power (along the *x* axis) and polarization (along the *y* axis), respectively. These multilevel conductivities can be attributed to the fractional volume of the GST that is switched along the nanowire (*22*); this, in turn, is dependent on the size of the laser spot that induces a temperature rise above the threshold. While we observe that the state of the nanowire is modulated linearly with respect to laser power (fig. S10), polarization-dependent switching causes more abrupt readout changes, because it is related to forming a conducting path, relying on a polarization-selective temperature rise above the threshold (fig. S5).

The above features enable freely accessible multilevels. As shown in Fig. 3D, by applying three consecutive polarization-controlled set (crystallization) and reset (amorphization) pulses (0°, 45°, and 90°) at the fixed power (7.5 and 30 mW, respectively), stepwise changes in current readouts are observed with the switching thresholds at around 45° polarization. The readout of our system depends on the solid state (which, in turn, modulates its resistivity) of our nanowires. This state is programmed using laser pulses. These states are nonvolatile and thus retained (i.e., stored) until the subsequent pulse. By increasing the fixed laser power for both set and reset processes, the threshold polarizations are shifted down to 0°. This polarization-controlled operation constitutes a nonlinear system, selecting the thresholds by polarization results in high-contrast multilevel outputs.

On the other hand, we observe linearly accessible levels when the power of the laser is modulated (8.5 to 12 mW) at a fixed polarization (Fig. 3E). At 45° polarization, the current readout gradually increases with each pulse and eventually gets saturated at 12 mW. When the polarization is set to 90° (s-polarized), 9.5 mW is large enough to saturate the readout. We find that the saturation current level remains unchanged at different polarizations (Fig. 3E) but varies with different laser powers (Fig. 3D). This underlines that saturation levels are determined by the total switched area (of the GST layer), which is dependent on incident laser power. These above results show a strategy for tailoring the threshold and saturation of nonvolatile readouts by polarization and power, respectively. Using these properties, the accessibility of polarization- and power-dependent multilevels in any random order can be achieved (see fig. S11). The observed discrepancies between the absolute values of the multilevel states can be further improved by optimizing the alignment and focusing of the laser.

**Fig. 4.**
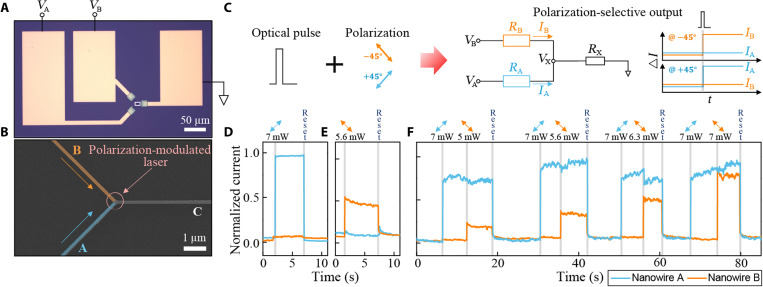
Polarization-selective demultiplexing. (**A**) Optical and (**B**) SEM images of multichannel encoding measurement. Nanowires A and B are oriented orthogonally and intersect at the junction. We attribute these as +45° and −45°, respectively. Each nanowire is biased independently (*V*_A_ and *V*_B_). Focused laser is illuminated onto the intersection. Scale bars, 50 and 1 μm. (**C**) Schematic of polarization-selective demultiplexing, where the optical pulses with +45° and −45° polarizations produce selective responses in resistances *R*_A_ and *R*_B_, thus *I*_A_ and *I*_B_. Simultaneous time-traced measurement of normalized electrical readouts from multichannels when (**D**) the +45° polarized pulse (7 mW) and (**E**) the −45° polarized pulse (5.6 mW) are sent. The readouts are initialized by a high-power amorphization pulse. (**F**) Reconfigurable and polarization-selective demultiplexing, where the 45° polarized pulses (cyan arrows) of the same laser power (7 mW) are applied, and, subsequently, the −45° polarized pulses (orange arrows) of reconfiguring laser powers (5 to 7 mW) are sent to set multiple weighting coefficients.

We now extend the concept of polarization-selective switching to demonstrating multichannel measurements of multiple HAD nanowires. This is done on a device that consists of two nanowires aligned orthogonally to each other (nanowires A and B in Fig. 4, A and B), intersecting at one end. A third nanowire (nanowire C) is connected to the ground. A voltage bias of 1 V is applied to each of the nanowires A and B, while the free terminal of nanowire C is kept at ground. We then illuminate the intersection of the three nanowires using the 638-nm laser and monitor the electrical current across nanowires A and B via nanowire C. We rotate the polarization from +45° and −45°, selecting only s-polarized nanowires to be switched (Fig. 4C).

Now, the resistance state of the nanowires A and B can be tuned selectively, although the laser illuminates both. Then, using different powers between 5 and 7 mW in increments of ~0.6 mW, we set the conductance levels (or weights) of each nanowire. As shown in Fig. 4D, when a +45° polarized pulse (7 mW) is applied to the system, this selectively crystallizes nanowire A (s-polarized) but not nanowire B (p-polarized). Similarly, a −45° polarized pulse (5.6 mW) only switches nanowire B, while nanowire A remains unaffected (Fig. 4E). Thus, only s-polarized nanowires respond to the input pulses, rendering the system capable of polarization-selective threshold operation.

We then verify the reconfigurability of polarization demultiplexing by independently encoding distinct weighting coefficients to both nanowires at the same time (Fig. 4F). We first selectively crystalize nanowire A using a +45° polarized pulse (7 mW; cyan arrows; Fig. 4F). Subsequently, a −45° polarized pulse with a smaller laser power (5 mW; orange arrows; Fig. 4F) is illuminated to switch nanowire B to the specific intermediate state. Thus, we use polarization to independently control each nanowire. By increasing the laser powers of −45° polarized pulses, we can selectively define five distinct levels of nanowire B without affecting nanowire A, therefore modulating the polarization-selective states more finely. This process is robust, and we found that nanowire A reliably reaches the same current level without affecting nanowire B when +45° polarized pulses of the identical laser power are applied in the same manner.

Note that, from the above results, our design exhibits active accessibility of micron-scale polarization-division multilevels with decoupled parameters, while the current state-of-the-art metasurface-based polarization-division (de)multiplexing operates in a passive or volatile manner (*8*, *29*, *30*). Not only do our results show reconfigurable encoding up to five different nonvolatile multilevels, but we also operate our device at tightly confined scales where the active switching area is ≤5 μm. Since the device footprint is dictated by the area of the focusing laser, the use of higher NA objective lens, in principle, can increase the resolution of the design and is ultimately limited only by the optical diffraction limit.

**Fig. 5.**
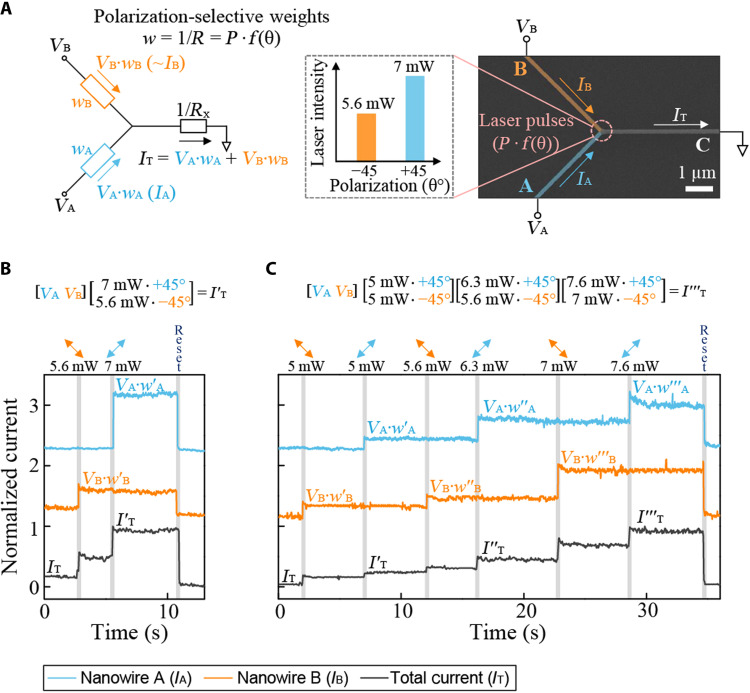
Matrix-vector multiplication using polarizations as the tunable vector. (**A**) Schematics of matrix-vector multiplication using polarization-selective weights. SEM scan with a scale bar of 1 μm. Each nanowire is subject to scalar multiplication between electrical input (*V*_A_ and *V*_B_) and synaptic weights (*w*_A_ and *w*_B_) to give output products (~*I*_A_ and ~*I*_B_), the sum of which results in *I*_T_. Synaptic weights refer to a parameter for conductance of each nanowire (1/*R*), and the load resistance (*R_x_*) is assumed to be smaller than the resistance of nanowire A and B (*R*_A_ and *R*_B_). The weights are dependent on laser power (*P*) at selective polarizations (θ). For the demonstration, 5.6 and 7 mW are used as examples for −45° and +45°, respectively. (**B**) Single and (**C**) multiple runs of matrix-vector multiplication operations. Cyan and orange curves represent the current through nanowire A (*I*_A_) and nanowire B (*I*_B_), respectively. Black curves represent total current (*I*_T_), flowing through to the third nanowire as shown in (A). Set pulses with varying laser power at polarizations of −45° and +45° are sent before the reset pulse.

The ability to program phase-change materials, such as the GST layer in our HAD nanowires, into multilevel states/weights has also been used in previous works to provide fast and efficient arithmetic, neuromorphic, and in-memory computing processing (*12*, *16*, *31*, *32*). We can also carry out such operations using HAD nanowire devices, but with the additional benefit of tunability/selection via the unique polarization sensitivity that we have demonstrated above. One arrangement for realization of such advanced processing functionality is shown in Fig. 5A, where we carry out in-memory multiply-accumulate (MAC) operations with the aid of polarization selectivity. Here, the electrical output of the total current (*I*_T_) through nanowire C is the sum of the inputs to nanowires A and B modulated by their relative weighting. In other words, each nanowire is subject to scalar multiplication between electrical input (*V*_A_ or *V*_B_) and the conductivity of the respective nanowire, which behaves as synaptic weights (*w*_A_ or *w*_B_). Figure 5B shows a single run of matrix-vector multiplication using this concept. We use polarization to selectively weigh each nanowire. Then, by modulating the currents at each nanowire, we measure the new output current *I*′_T._ We are able to carry out multiple MAC operations by updating the weights deterministically, as shown in Fig. 5C. As we show, we update these weights to subsequently measure new outputs *I*″_T_ and *I*″′_T_. These operations can be continued until either the entire active region of GST becomes crystallized or a reset pulse is applied. Although the weights are monotonically increased here, we can use double-step pulses (*22*) or fine-tuned reset pulses to randomly program them in any order (fig. S11). In addition, by further optimizing the geometry (fig. S12) of nanowire C or replacing it with a metal, we can minimize any undesired switching and facilitate an interconnection to the next computing unit for cascadable systems (*33*, *34*).

Our work allows multiple MAC operations on a micron-sized element using a free-space laser. Such compact MAC operation is achieved, because the elements are selected by polarization-specific and confined resonances in nanowires. This enables effectively reconfiguring distinct elements with simple polarization controls, which is different from devices using ring resonators (*12*) or directional couplers with Mach-Zehnder interferometers (*35*) that use wavelength- or phase-dependent selectors. The spatial resolution of our design can be further enhanced by assembling more nanowires (see figs. S13 and S14). By simultaneously carrying out multiple operations over independent polarization channels, our system shows the potential for ultrahigh-speed modulation, surpassing the computing density of digital electronics (*36*, *37*) by several orders of magnitude (table S1). Further design optimization is required for cascaded computing units in a large-scale system. Moreover, hybrid nanowires are also wavelength dependent as a function of the widths of nanowires (fig. S1), which is another parameter to explore for multiplexing optical information and maximizing computational density. By extending this concept beyond linearly polarized light, tunable optoelectronics/photonics with vortex beams can also be achieved that exploits orbital angular momentum (*38*, *39*) or optical chirality (*40*, *41*) as a tunable factor.

## DISCUSSION

In conclusion, we demonstrate a HAD structure in a nanowire configuration that allows us to use polarization to selectively address them. We use GST, a common phase-change material; we choose where the GST undergoes a phase change by using polarization as a variable. Our unique approach uses dielectric resonances to both select and switch absorptive active elements. This is the first proof of concept for polarization-selective switching, incorporating tunability in a HAD nanostructure. Using this approach, we demonstrate reconfigurable and nonvolatile polarization-division demultiplexing of electrical conductivity with up to five independent levels. We extend this electrically coupled and polarization-decoupled design to demonstrate matrix-vector multiplication (MAC-type operations) with input polarization as the tunable vector element. Our results unlock an additional degree of freedom in phase-change photonics and pave the path for a breadth of applications that fully exploit multifaceted properties of light.

## METHODS

### Device fabrication

A first run of standard electron beam lithography (EBL) was performed on doped 525-μm Si wafers with a 330-nm SiO_2_ layer to fabricate the patterns for metal electrodes. Cr and Au of 5 and 50 nm thickness were sequentially deposited onto the patterned substrate via thermal evaporation, and a subsequent lift-off process was carried out. A second step of EBL was performed to define the nanowires with widths of ~180 nm and the contacts with sizes of ~10 μm × 15 μm between the prefabricated metal electrodes. The patterned substrate was subjected to another lift-off process, sequentially followed by deposition of GST and Si layers of 15 and 65 nm thickness, respectively, via radio frequency (RF) sputtering system (Nordiko). Both layers of EBLs were done on a positive photoresist (CSAR62). Prior to the measurement, the fabricated devices were placed on a hot plate (200°C, 5 min) to fully crystallize the GST and improve the contact resistances.

### Measurement setup

The electrical and optical measurement of electro-optic cells was acquired using a customized microscope setup with electrical probes, as shown in Fig. 2A. The linearly polarized laser (Vortran Stradus 639) was modulated by a pulse generator (TG5011) from nanoseconds to microseconds, which were used to switch crystalline phases of the nanowires. The pulsed light was propagated through an HWP to adjust the polarization directions and then illuminated onto the device through a 0.28-NA objective lens. For reliable switching processes, laser light was slightly defocused to obtain more uniform power distribution and therefore minimizes the damage at the center of laser spots. The final optical powers were measured by rotating the HWPs to confirm that power was consistent while polarization rotated (fig. S9). We considered ±1.5 mW fluctuation in setting the laser power due to slight misalignment of focused spot. Laser power was measured using an optical power meter (Thorlabs, PM100D) in the same setup. The current output of the devices was continuously monitored by a source meter unit (SMU; Keithley 2614B) by applying a DC source voltage of 1 ± 0.4 V to two ends of metal electrodes via metallic probes. Dual channels of SMU were used for simultaneous time-traced measurement of multichannel currents, with the same applied voltages.

### Modeling

Numerical simulations were carried out with a commercial FDTD software package (Lumerical FDTD, Lumerical Solutions) to calculate absorption/reflection spectra, electrical field, and absorbed power distribution of hybrid nanowires for a 2D model with a plane wave source. The spectral response of a single-element nanowire was obtained by considering it with excessively large periods (i.e., 2 μm). We also perform 2D FEM thermal simulations (COMSOL Multiphysics) to explore the electromagnetic heating of hybrid nanowires under plane-wave illumination by varying polarization directions, laser powers, or pulse duration. The dielectric function for GST and Si from hybrid nanowires was measured from ellipsometry results on a thin film prepared by RF sputtering. Those of SiO_2_ and Si from substrates are obtained from Palik (*42*). The heat capacity, thermal conductivity, and density of GST are obtained from previous studies (*43*, *44*), while their size-dependent thermal properties (*45*) were not considered.
