# Supplementary material for: Polarization-selective reconfigurability in hybridized-active-dielectric nanowires
Source: Sci Adv. 2022 Jun 15;8(24):eabn9459. doi: 10.1126/sciadv.abn9459 (PMC9200283; doi:10.1126/sciadv.abn9459)
Supplement: Supplementary file 1 — Supplementary Text Figs. S1 to S14 Table S1 [file sciadv.abn9459_sm.pdf]

Science Advances
AAAS

Supplementary Materials for

Polarization-selective reconfigurability in hybridized-active-dielectric nanowires

June Sang Lee *et al.*

Corresponding author: Harish Bhaskaran, harish.bhaskaran@materials.ox.ac.uk

Sci. Adv. **8**, eabn9459 (2022)
DOI: 10.1126/sciadv.abn9459

This PDF file includes:

Supplementary Text
Figs. S1 to S14
Table S1

Spectral responses of hybrid nanowires as a function of wavelength and polarization directions

We present the calculated maps of reflection and absorption spectra for different types of nanowires (bare Si, Si/aGST, and Si/cGST) as a function of nanowire widths (Fig. S1) and incident polarization angles (Fig. S2). We consider boundary (≥2 μm) in the simulation to ensure the absence of mode overlap between adjacent elements and to explore the spectral responses of a single nanowire. The dielectric nanowire resonances are found to be produced in a hybridized form with additional thin-film interaction at 330 nm-thick SiO_2 layer of substrates. As expected, we observe the red-shifted reflection dips (dashed lines) as s-polarized nanowire widths are increased with suggesting that multipolar excitations are confined within the nanowire. The stationary reflection dip at 600 nm despite the change of nanowire widths, depicts the Fabry-Perot resonances from the SiO_2 thin film. Such reflection dips are mostly translated into the absorption peaks for all three scenarios (Fig. S1b). The Si cavity is considered for absorption calculation in Fig. S1b (i) and S2b (i), but the GST layer only is considered for calculation in Fig. S1b (ii, iii) and S2b (ii, iii). More complex multipolar modes are simultaneously generated for the ones with extra GST layers (ii, iii). Then, as shown in Fig. S2, the hybrid nanowires with widths of 180 nm exhibit polarization-dependent reflection/absorption changes, as their spectral properties stay close to the resonant wavelength. As the incident polarization angle becomes 90°, reflection decreases and absorption increases due to more dominant dielectric excitations under s-polarized illumination. Since the operating wavelength (638 nm) of our devices are within the higher-order mode resonances of nanowires (630 – 660 nm), the nanowires exhibit polarization-sensitive absorption, thereby enabling polarization-sensitive resonant switching.

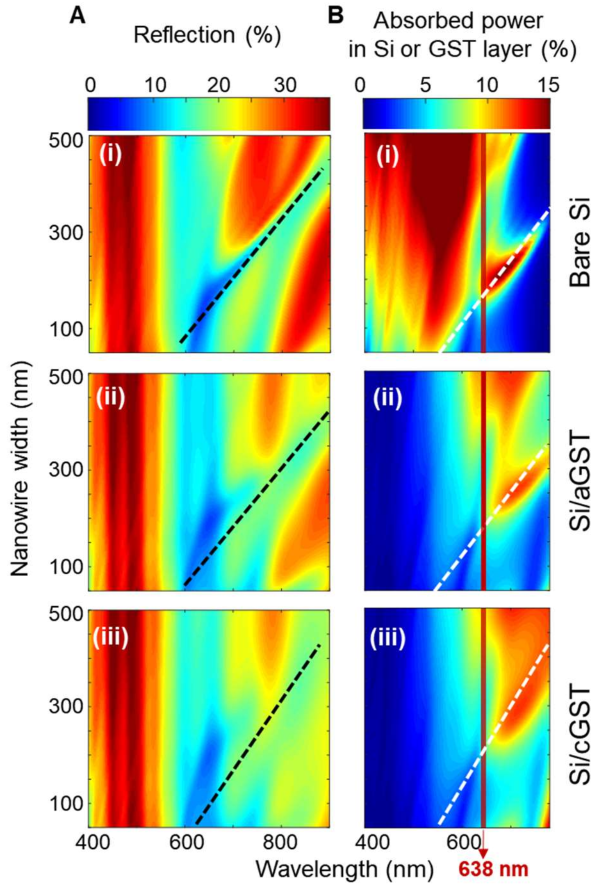

Fig. S1. Spectral responses of nanowires as a function of varying widths. Calculated maps of **(A)** reflection and **(B)** absorption spectra as a function of varying nanowire widths, for bare Si (i, top), Si/aGST (ii, middle) and Si/cGST (iii, bottom) configurations under s-polarized illumination. Dotted lines depict hybridized dielectric resonances of nanowires. For absorption, only Si or GST layer is considered for each calulation in (i, bare Si) and (ii, Si/aGST and iii, Si/cGST), respectively.

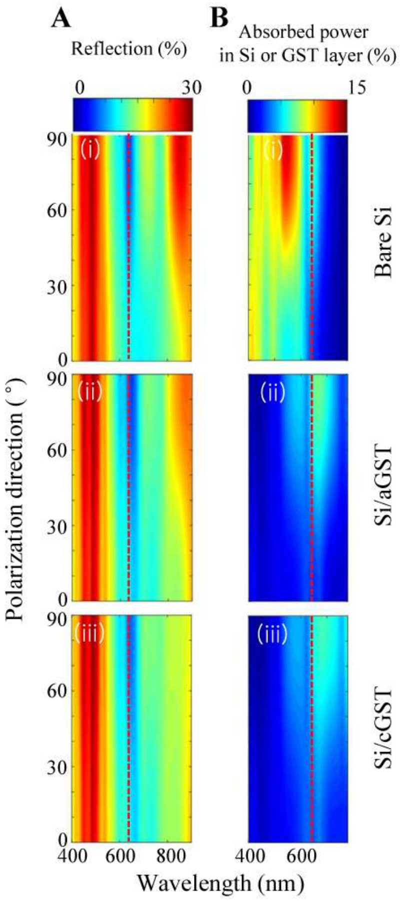

Fig. S2. Spectral responses of nanowires as a function of varying polarization angles. Calculated maps of **(A)** reflection and **(B)** absorption spectra as a function of varying polarization angles from 0° (p-polarized) to 90° (s-polarized), for bare Si (i, top), Si/aGST (ii, middle) and Si/cGST (iii, bottom) configurations with a nanowire width of 180 nm. Red dotted lines show the laser line at 638 nm. For absorption, only Si or GST layer is considered for each calulation in (i, bare Si) and (ii, Si/aGST and iii, Si/cGST), respectively.

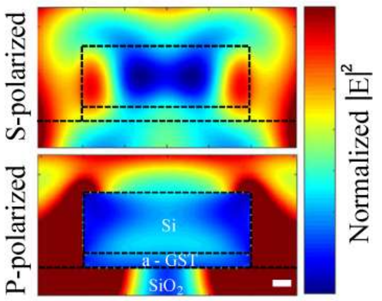

Fig. S3. Cross-sectional views of electric-field distribution. Laser at the wavelength of 638 nm is illuminated onto s-polarized (top) and p-polarized (bottom) nanowires. The intensity of the electric-field is normalized for both polarizations. Scale bar is 20 nm.

2D FEM simulation of hybrid nanowires

The heat dissipation, mainly generated from the highly absorbed regions of the GST, allows temperature rise to be uniformly spread out across the cross-sections as the pulse length becomes long enough for each phase-switching operations (i.e. 1 μs and 80 ns), as depicted in Fig. S4-5. In Fig. S4a and S4c, when pulse duration is much shorter than the required switching time (i.e. 5 ns), one can see that cross-sectional views of temperature profile are analogous to absorption profile of the GST in Fig. 1C. However, after the required switching time (1 μs and 80 ns) in Fig. S4b and S4d, the temperature becomes uniformly distributed across the GST cross-section and above switching temperatures (493 and 900 K, respectively). Also, such uniformly distributed temperature varies as a function of input polarization angles (Fig. S5) with following the sinusoidal-like increase of averaged temperature and optical absorption (Fig. 2B, C). We note that these results are not related to multilevel operation as a function of pulse duration.

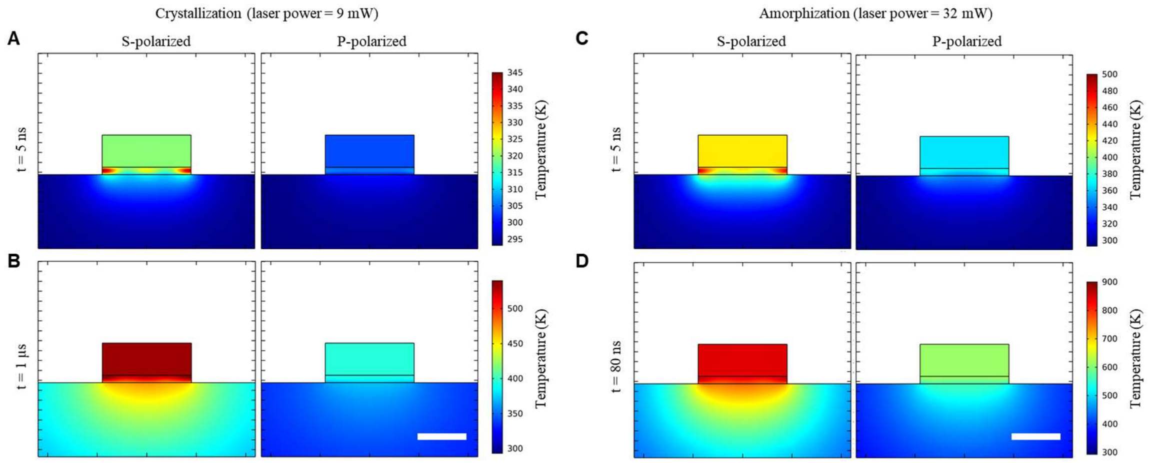

Fig. S4. Cross-sectional view of tempearture profile within hybrid nanowires. The temperature profiles are illustrated for **(A-B)** crystallizaiton processes and **(C-D)** amorphization processes, where the laser power (pulse durations) are 9 mW (1 μs) and 32 mW (80 ns), respectively. Left (right) panels of each profile is s-polarized (p-polarized) nanowires. Top panels show the temperature profile when the time taken from the pulse illumination is 5 ns, and bottom panels show when the pulse is just turned off after the corresponding pulse durations (i.e. 1 μs for crystallization and 80 ns for amorphization). Scale bar is 100 nm.

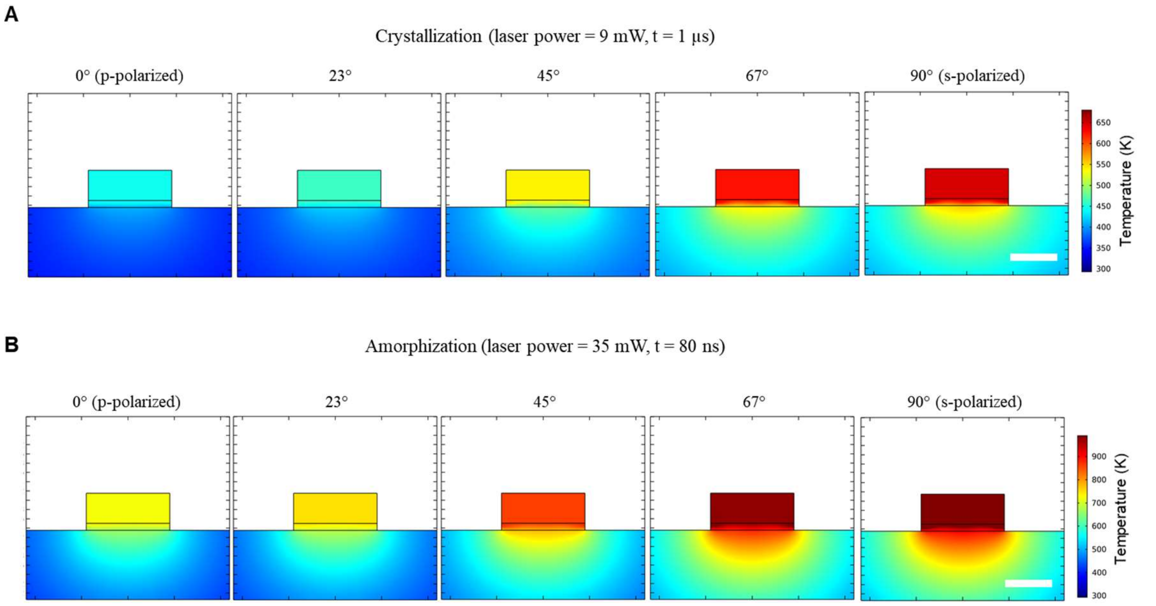

Fig. S5. Cross-sectional views of temperature profiles at each polarization angle. Laser power (pulse duration) is 9 mW (1 µs) and 35 mW (80 ns) for **(A)** crystallization and **(B)** amorphization processes, respectively. Scale bar is 100 nm.

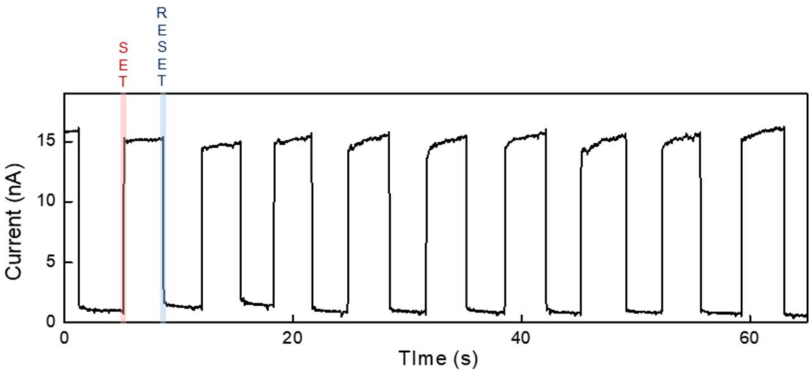

Fig. S6. Reversible switching of the nanowire with electrical readout measurement. SET and RESET represent optical pulses of 9 mW at 1 µs and 35 mW at 80 ns, respectively, at a fixed polarization angle of 90° (s-polarized).

Multilevel operation dependent on light intensity

Distinct multilevels are achieved due to the fractional volume of the GST that exhibits temperature rise above the threshold (T_g (glass transition temperature) and T_m (melting point)). In our case, it is specifically related to how far away from the centre of the laser spot, the threshold temperature is reached, and this temperature profile varies as a function of the input light intensity (laser power (Fig. S7) or pulse duration (Fig. S8)).

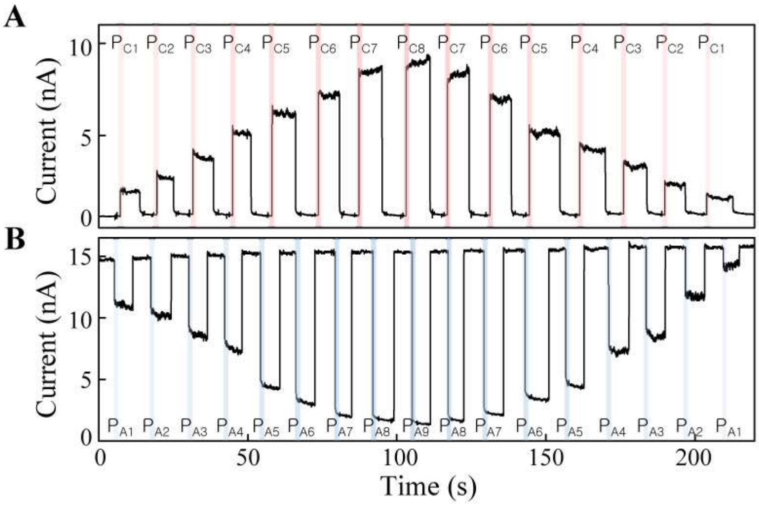

Fig. S7. Power-dependent multilevel electrical readouts. P_{C1} – P_{C8} represent optical pulse power from 5 to 10 mW at fixed pulse duration of 1 µs for **(A)** crystallization processes, and P_{A1} – P_{A9} represent 26 – 37 mW at fixed pulse duration of 80 ns for **(B)** amorphization processes. The identical initialization pulses (RESET for **(A)**, SET for **(B)**) are applied after each programming pulse.

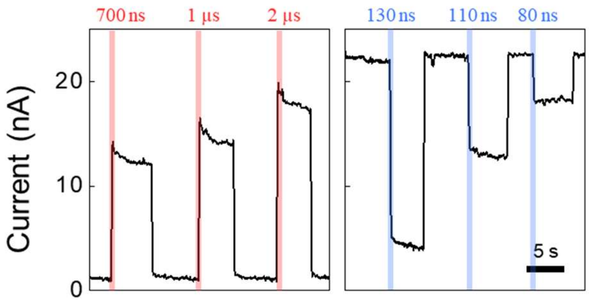

Fig. S8. Pulse duration-dependent multilevel electrical readouts. The power is 11mW (left, crystallization) and 25 mW (right, amorphization), and the polarization angle is fixed at 90° (s-polarized).

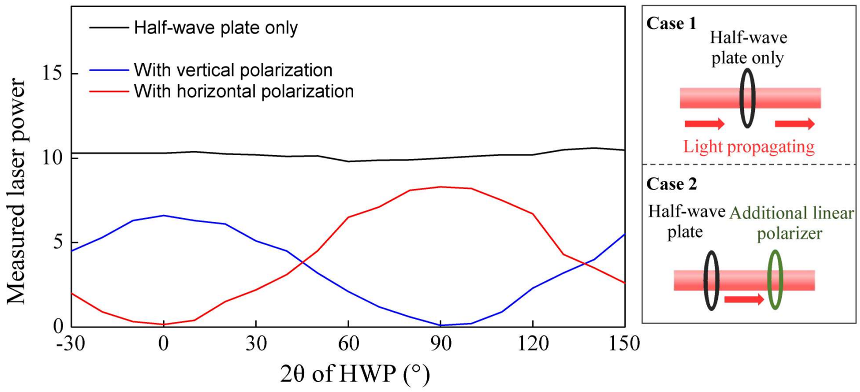

Fig. S9. Measured laser power as rotating the angle (θ) of half-wave-plate (HWP). Schematics show the configuration of optical components for each measurement. Black lines refer to the power measurement with HWP alone inserted in. Red and blue lines refer to the power measurement with additional linear polarizer at 0° (blue, vertical) and 90° (red, horizontal) angles, respectively.

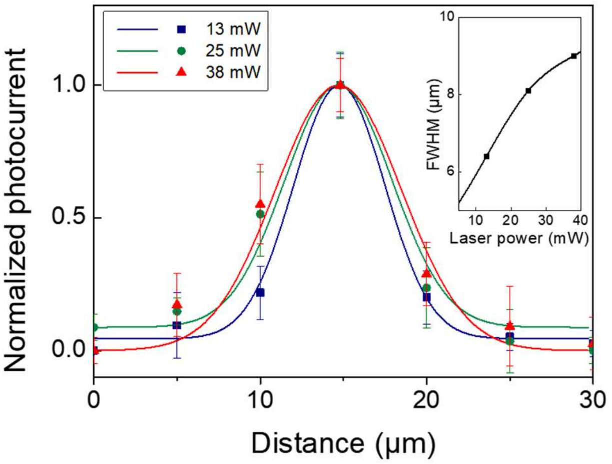

Fig. S10. 1D normalized photocurrent profile and Gaussian fitted curves along the scanning distance at different CW laser powers. The measured full-width-half-maxima (FWHM) of each curve is 6.4 μm (blue), 8.1 μm (green) and 9.1 μm (red), which is estimated to approximate the laser spot sizes. Inset images show the polynomial-fitted trend of laser-spot sizes as a function of laser power. The actual switching area can be smaller than the measured spot sizes, depending on laser pulse lengths.

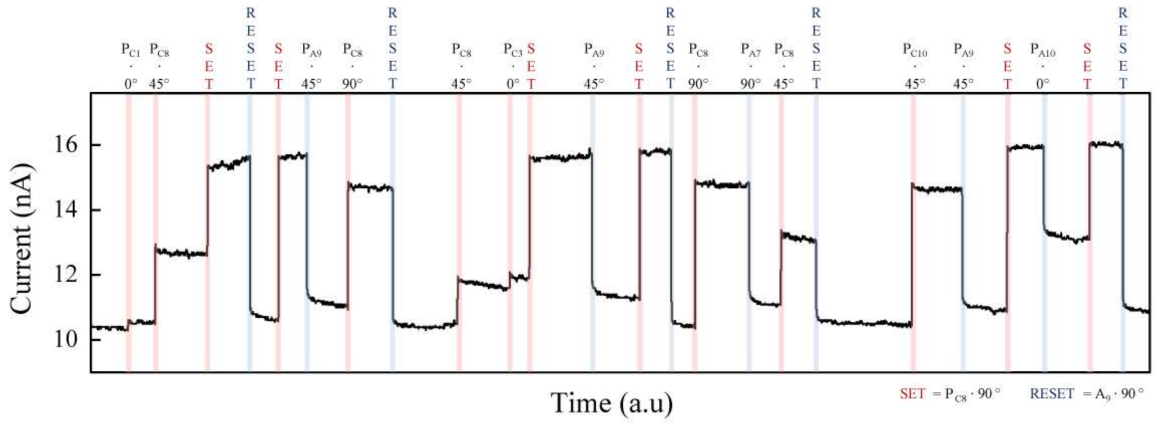

Fig. S11. Time-trace electrical readouts of multilevel operations under randomly programmed pulses. Each notation represents laser powers for crystallizaiton (P_{C1} (5mW) - P_{C8} (10 mW)) and amorphization (P_{A7} (34 mW) - P_{A10} (38 mW)), and polarization angles (0°, 45°, 90°).

Optimization of the output nanowire

In Fig. 5, a third nanowire (nanowire C) is used as an output channel to sum up the electric current from the other operational nanowires (nanowire A and B). During the operation, it is important to keep the state of nanowire C always constant so that undesired switching is suppressed, and we only observe the change of conductivities in nanowire A and B. To ensure this selectivity, we can further tailor the absorption of nanowire C by decreasing its width. As shown in Fig. S12, one can note that the 90 nm-wide nanowire (i.e. nanowire C) shows lower absorption than the 180 nm-wide nanowire (i.e. nanowire A and B). Therefore, nanowire A and B are polarization-selectively switched upon s- and p-polarized pulses of incoming light, while the nanowire C (that is aligned at 45° to the input polarization angles) always remains unaffected.

However, a narrower width of nanowire C can give rise to higher resistivity; the resistivity of nanowire C should be always lower than the one of nanowire A and B. We can overcome this by using metallic materials (eg Al or Au) for nanowire C.

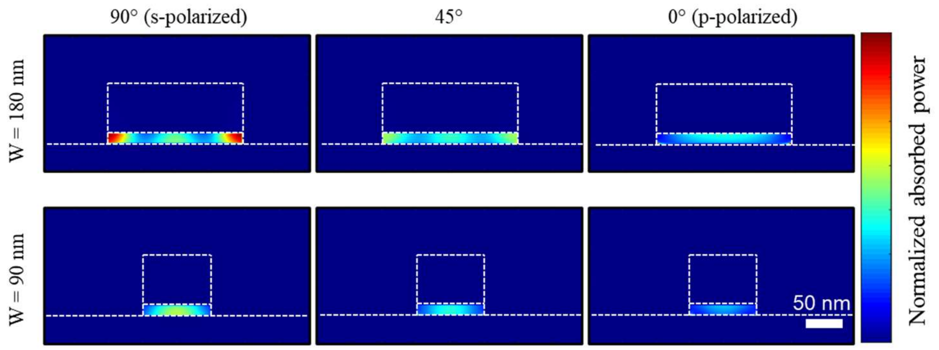

Fig. S12. Cross sectional view of FDTD simulations of absorbed-power distribution for different nanowire widths. Nanowire widths of (top) 180 nm and (bottom) 90 nm are used at incident polarizations of (left) 90°, (middle) 45°, and (right) 0°. Scale bar is 50 nm.

Polarization-selective performance depending on nanowire lengths

Polarization-selective absorption contrast depends on aspect ratio of nanowire lengths to widths. As shown in Fig. S13, we calculate the absorption of nanowires at both polarization directions with changing the nanowire lengths from infinity to 500 nm, while the nanowire width is fixed at 180 nm. We observe that polarization-selective absorption is found to be maintained at the nanowire length (aspect ratio) of 500 nm (2.7:1). This provides additional opportunity to scale down the electro-optic device, thus increase the device densities.

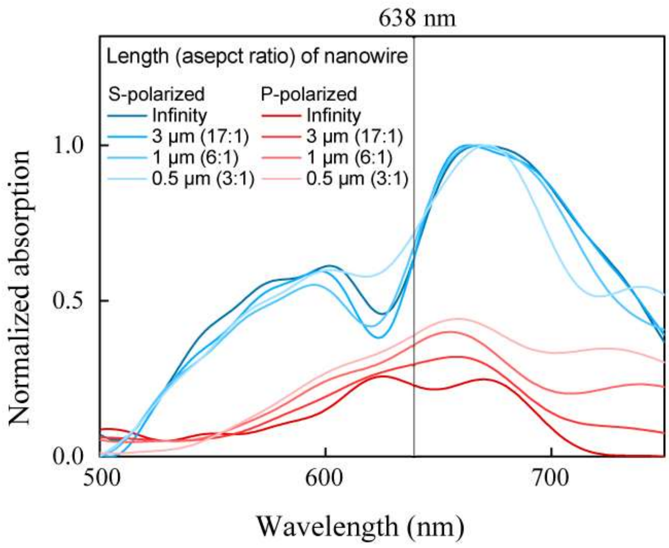

Fig. S13. Normalized absorption spectra for s- and p-polarized nanowires with different lengths.

Compact polarization-selective electro-optic devices

As shown in Fig. S14, by decreasing the input laser power below ~6.5 mW, the threshold polarization angle to crystallize the nanowire is above 45°, where 0° and 90° represent p- and s-polarized conditions, respectively. Therefore, the incoming light at 45°-polarization cannot switch the nanowire and this allows more nanowires to be embedded at different orientations in a single device. For instance, four nanowires can be assembled as shown below, where each nanowire is 45° apart from each other. Therefore, the device density can be further increased by incorporating multiple nanowires under the same illumination area.

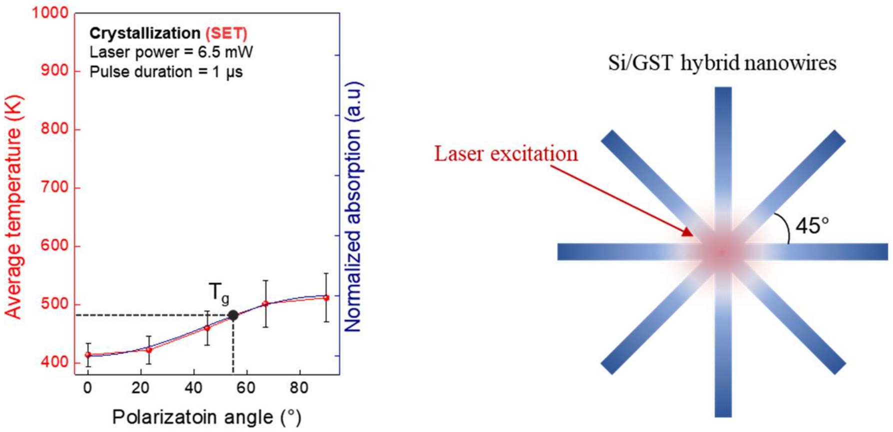

Fig. S14. Calculated temperature and optical absorption of a nanowire as a function of polarization angles at low laser power. (Left) Calculated averaged temperature and normalized absorption within the GST of a hybrid nanowire as a function of polarization angles for crystallization at low power of 6.5 mW. T_g refers to a glass transition temperature of GST. (Right) Schematic diagram of compact multi-polarization-addressable nanowires with the angles of 45° apart from each other

Compute density comparison

The quantitative evaluation of compute density between our device and current electronic and photonic architectures is shown in Table S1. We use TOPS (trillions of operations per second)/area, a well-known figure-of-merit, to analyze the compute density as demonstrated in the following reference (*Nature*, **589**, 52, (2021)) (*16*). In our case, clock speed is roughly estimated as the switching speed of the crystallization process and the computing area is assumed to be the size of a laser spot or a multiple-nanowire configuration.

As demonstrated in Table S1, the compute density of our system is found to be several orders of magnitude larger than those of electronics (*37, 38*) and comparable to the one of photonic in-memory computing with wavelength-division multiplexing (WDM). Our system possesses an additional functionality that can selectively tune the synaptic weights. Because such compute density is largely dependent on the size of device footprints, one can find that this can be dramatically enhanced by decreasing the size of computing units (Fig. S13) or assembling more nanowires (Fig. S14). However, further design optimization is required for simultaneous illumination on the multiple computing units and their cascadabilities in a large-scale system.

	Multiplexing parameter	Clock speed (GHz)	Number of operations per time step	Footprint of a MAC unit (μm)	Selectively tunable synaptic weights	Compute density (TOPS/mm^2)	Ref.
This work (experimental)	Polarization	1[a]	2	~21 × 25[c]<br>~5 × 5[d]	Yes	3.8<br>80	-
This work (theoretical)	Polarization	1[a]	4[b]	0.5 × 0.5[e]	Yes	16000	-
Nature (2021)	Wavelength	12, 18, or 25	16	285 × 354 (SiN)[f]<br>30 × 30 (SOI)[g]	No	1.2 (SiN)<br>162 (SOI)<br>880 (future scaling)[h]	(*16*)
Haswell E5-2699 v3	-	2.3	1	-	-	0.0039	(*37*)
Google TPU (ASIC)	-	0.7	1	-	-	0.28	(*37*)
Nvidia Tesla P40 (GPU)	-	1.3	1	-	-	0.1	(*38*)

Table S1. Computing density comparison with current electrical and optical devices. [a]Switching speed of a crystallization process. [b]Number of polarizations that can be used in a single computing unit (from FDTD calculation in Fig. S14). [c]Size of multiple-nanowire configuration (from Fig. 5). [d]Size of a laser spot at the laser power of 5 mW (from Fig. S10). [e]The minimum required size of nanowire to exhibit polarization-selective switching (from FDTD calculation in Fig. S13) within optical diffraction limit when using a high NA lens. [f, g]Size of each computing unit for silicon nitride (SiN) and silicon-on-insulator (SOI) platform. [h]Calculated computing density for the upscaled design. Note that the area of active region or photonic tensor core itself is considered for our system and the convolutional photonic architecture from the reference (*16*), respectively.
